# Harm Perceptions of the JUUL E-Cigarette in a Sample of Ever Users

**DOI:** 10.3390/ijerph17134755

**Published:** 2020-07-02

**Authors:** Elise M. Stevens, Emily T. Hébert, Alayna P. Tackett, Eleanor L. S. Leavens, Theodore L. Wagener

**Affiliations:** 1 Harvard T.H. Chan School of Public Health, Dana-Farber Cancer Institute, Harvard University, Boston, MA 02115, USA; 2 Oklahoma Tobacco Research Center, Stephenson Cancer Center, University of Oklahoma Health Sciences Center, Oklahoma City, OK 73104, USA; Emily-hebert@ouhsc.edu (E.T.H.); Alayna-tackett@ouhsc.edu (A.P.T.); 3 Department of Population Health, University of Kansas Medical Center, Kansas City, KS 66160, USA; eleavens@kumc.edu; 4 Center for Tobacco Research, The Ohio State University Comprehensive Cancer Center, Columbus, OH 43210, USA; Theodore.Wagener@osumc.edu; 5 Department of Internal Medicine, The Ohio State University, Columbus, OH 43210, USA

**Keywords:** JUUL, electronic cigarettes, tobacco products, harm perceptions

## Abstract

Background: Monitoring trends and perceptions of new nicotine salt-based electronic cigarettes (ECs), like JUUL, is important to identify associations with product experimentation and use. Understanding harm perceptions of these new devices will inform prevention and intervention efforts. The current study assesses perceptions of the absolute harmfulness of JUUL use in addition to comparing it to other tobacco products. Methods: Participants (*N* = 839, 52% male) reporting ever use of JUUL were recruited from Amazon’s Mechanical Turk from January to March 2018. Respondents completed questionnaire items assessing demographics, co-use of non-JUUL products, JUUL use status (i.e., daily users (10.8%), non-daily users (29.4%), and triers (59.9%)), and JUUL and other tobacco products absolute harm perceptions. Results: Overall, participants rated JUUL as significantly less harmful than all other tobacco products (*p* < 0.001), except other ECs. Daily JUUL users rated JUUL as less harmful compared to non-daily JUUL users and JUUL triers (*p* < 0.05). JUUL was rated as more harmful by women compared to men (*p* < 0.05). Conclusions: Increased frequency of JUUL use was associated with decreased harm perceptions. JUUL was associated with reduced perceptions of absolute harm compared to most other tobacco products, except other ECs. Public health practitioners should develop public health interventions that increase harm perceptions of ECs.

## 1. Introduction

As combustible cigarette use rates continue to decline [1], the use of electronic cigarettes (ECs) is on the rise [2]. In the United States, 2.8% of the population use ECs, which translates to 6.9 million people [3]. The popularity of ECs may be due, in part, to the introduction of nicotine salt-based products, such as JUUL [4]. Since its introduction in 2015, JUUL has taken over the EC market accounting for over 70% of the share [5]. Most worrisome about its popularity is its prevalence among those who are tobacco naïve and are not looking to quit smoking in favor of a less harmful alternative [6]. While research shows that JUUL and other EC use may have less negative health consequences than combustible products [7,8], e-cigarette aerosol is not harmless, and it is important to understand harm perceptions of the product as they may impact the use of JUUL [9] and possibly the progression of using more harmful products, such as cigarettes [10,11,12,13].

JUUL is shaped like a USB flash drive and has simple, switchable pods filled with 3–5% nicotine salts. The e-liquids come in tobacco and menthol flavors and can deliver nicotine to the user at levels similar to that of a combustible cigarette [14,15]. According to the company, each JUUL pod is equivalent to 200 puffs of a combustible cigarette or one pack of combustible cigarettes (from www.juulvapor.com). Unlike tank or mod e-cigarettes, JUUL is easily concealable [16], which likely facilitates use in places were traditional tobacco use is prohibited.

Limited research exists on the actual health effects and perceptions of harm of JUUL. However, some research on the health effects of JUUL show that formaldehyde and total aldehyde yields were lower in JUUL compared to other studied ECs [17]. However, JUUL was shown to have a very high nicotine content [17], which is likely to lead to nicotine addiction and dependence [18] and could lead to the eventual use of combustible cigarettes [10,11,12,13]. Research about JUUL-related harm perceptions is also sparse. Studies show that youth and young adults that use JUUL perceive ECs, in general, to be less harmful than combustible cigarettes and may have a greater addiction potential [18]. One other study showed that young adults perceived a 40% chance of experiencing short- and long-term health risks from using pod-mod ECs like JUUL [19]. Two recent studies specifically examined JUUL-related harm perceptions and showed that individuals believed that JUUL use would lead to some harms [20] and that flavors could lead to increased harm [21]. However, these studies were limited to only the perceptions of adolescents. While these studies shed light on JUUL’s potential for harm, they did not examine harm perceptions of JUUL among adult users of the product. Research has shown that, in general, harm perceptions of ECs impact initiation and use of ECs [22]. In fact, the majority of adults believe ECs are less harmful than combustible cigarettes [23,24,25,26]. Interestingly, adults who were followed over time in a longitudinal study showed that those who perceived ECs as less harmful and socially favorable were more likely to initiate use [27]. However, these studies did not evaluate harm perceptions specific to JUUL.

Because JUUL has caused such a shift in the EC market, it is imperative to understand how users perceive the product harmfulness and how those perceptions may differ based on demographic factors (e.g., sex, race/ethnicity, age) as well as JUUL use. We hypothesize that JUUL ever-users will perceive the product as less harmful than combustible cigarettes and other types of ECs. Additionally, consistent with existing studies of other tobacco products, we anticipate that greater experience with the product will be associated with decreased perceptions of harm [28]. Using an online survey, the current study examined absolute harm perceptions of JUUL and other tobacco products among varying types of JUUL users (i.e., daily, non-daily, and triers) in adults 18 years and older. By understanding these associations, researchers and public health officials can be informed about the ways in which the product is perceived to assist in the design of more effective interventions, public health campaigns, and other prevention efforts.

## 2. Materials and Methods

### 2.1. Participants

Participants were recruited from Amazon’s Mechanical Turk (MTurk), an online crowdsourcing platform [29], from January to March 2018. To complete the survey, participants self-selected to be in the study entitled “E-Cigarette Use Patterns.” After providing informed consent, participants were screened for eligibility. Eligible participants were (1) 18 years or older, (2) endorsed ever use of JUUL, and (3) had an IP address from the United States. Screening was masked by asking other EC and health behavior questions. If eligible, participants completed the survey via Qualtrics and then were compensated $1–2 via MTurk in January through March 2018. All procedures were approved by the University’s Institutional Review Board (#AS17102).

### 2.2. Measures

Demographics. Participants were asked about age, race/ethnicity, and biological sex.

Use of nicotine products. Participants were provided with an image of each of the following tobacco products to enhance clarity and accurate reporting: JUUL, cigarettes, smokeless tobacco, snus, hookah/waterpipe, ECs (cig-a-likes, tanks, mods), cigars, cigarillos, small cigars, pipe tobacco, dissolvable tobacco. Ever use of JUUL was required for inclusion in the survey. For other products, participants were asked “Have you ever used [*insert product*]?” with response options of “yes,” “no,” and “I don’t know.” Participants who answered “yes” to each product were asked “How often do you currently use [*insert product*]?” from a modified scale from the Adult Population Assessment of Tobacco and Health (PATH) [30]. The current measure was modified in such a way that gave participants more specific use pattern options. For instance, the PATH scale offers only use choices such as “every day,” “some days,” or “not at all.” The responses in this study included: (1) “Daily or almost daily, (2) “Less than daily, but at least once a week,” (3) “Less than weekly, but at least once a month,” (4) “Less than monthly,” (5) “Not at all,” and (6) “I don’t know.” Use of each product was recoded to (1) never-user, if the participant had never tried the product, (2) daily, if the participant used the product daily, (3) non-daily, if the participant used the product between once a week and once a month, and (4) trier, if the participant used the product less than monthly or not at all. Responses of “I don’t know” were categorized as missing. Participants who reported using any of the non-JUUL products at least once a month were classified as “current users of at least one non-JUUL product.”

Absolute harm perceptions. Participants were to rate their absolute perceived harm of JUUL and each tobacco product (i.e., cigarettes, smokeless tobacco, snus, hookah/waterpipe, ECs (cig-a-likes, tanks, mods), cigars, cigarillos, small cigars, pipe tobacco, dissolvable tobacco) separately, “On a scale from 0 to 10, how harmful is [tobacco product]?” In a similar fashion, we also examined harm perceptions of the United States’ Food and Drug Administration (FDA) -approved methods of nicotine delivery (nicotine replacement products). Scales were taken from past work used to measure EC harm perceptions [24].

### 2.3. Data Analysis

Descriptive statistics were used to examine participant demographics and the average perceived harm rating of each product. To compare the average perceived harm by product, a one-way between subjects’ ANOVA with a Tukey post-hoc test was conducted. To assess whether perceived harm of JUUL was associated with JUUL use status, a regression analysis was performed, adjusting for age, race, biological sex, and use of other non-JUUL products. Studentized residuals were tested and assumptions of normality were met. JUUL use status was entered as the independent variable. All analyses were conducted in IBM SPSS Statistics, version 25 (IBM, Armonk, NY, USA).

## 3. Results

### 3.1. Participants

Participants (*N* = 839) were mostly male (52.1%), white (75.5%), and were on average 33.3 years old (SD = 9.3). Most participants had tried JUUL but used it less than monthly (triers, 59.9%), 29.4% used JUUL less than daily but at least once a month (non-daily users), and 10.8% used JUUL daily.

### 3.2. JUUL and Other Tobacco Use Patterns

Participants were categorized by frequency of use with less than monthly categorized as triers (59.9%). Over a quarter (29.4%) used JUUL less than daily but at least once a week (non-daily users). Lastly, 10.8% used JUUL daily (daily users), which differs from probability-based estimates. Table 1 illustrates tobacco use status of each product for the sample. Of the sample, the largest daily users were for cigarettes followed by modified ECs, tank ECs, and JUUL.

### 3.3. Absolute Harm Perceptions

**Figure 1:**
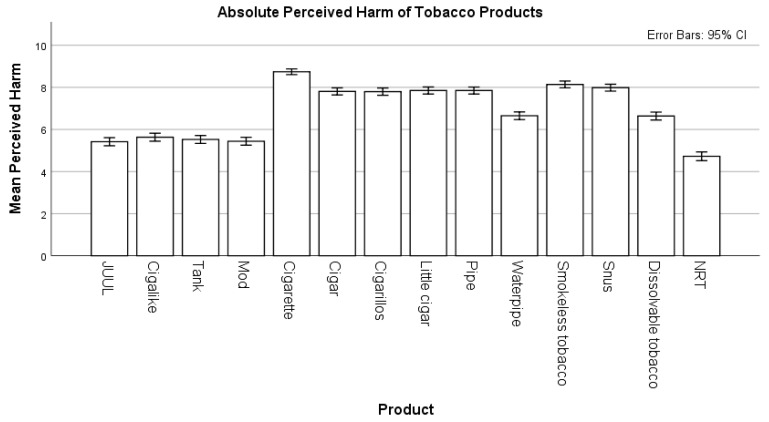
Absolute perceived harm of tobacco products and nicotine replacement therapy compared to the absolute perceived harm of JUUL. Absolute perceived harm was measured on a scale from 0 (not harmful at all) to 10 (extremely harmful).

Participants rated JUUL as similarly harmful as other e-cigarettes (i.e., cigalike, tank, and modified tank ECs), but significantly less harmful than all other tobacco products (*p* < 0.001). Compared to JUUL, the FDA-approved nicotine replacement products were rated lower in absolute harmfulness (*p* < 0.001). The average perceived harm of each tobacco product is described in Figure 1 and Table 2.

The regression model examining the relationship between perceived harm of JUUL, use of non-JUUL nicotine products, and JUUL use status is shown in Table 3. JUUL use was significantly associated with perceived harm of JUUL, such that lower perceived harm was associated with increased frequency of JUUL use (*p* < 0.05). Race was not associated with perceived harm of JUUL; however, sex was significantly associated with perceived harm, with JUUL rated as more harmful among women than among men (*p* < 0.05). Use of at least one other product that was not JUUL was also significantly associated with perceived harm of JUUL, with those who used other products rating JUUL as less harmful than those who exclusively used JUUL.

## 4. Discussion

This study examined the harm perceptions of what is considered the most popular EC on the market, JUUL, among adult users of the product. The results indicate that most ever JUUL users were triers (less than monthly); however, 40% were non-daily or daily users. JUUL was rated as less harmful than all other tobacco products except tank, modified ECs, and cigalikes, but was rated as more harmful than the FDA-approved nicotine replacement products. Men, and those who used JUUL more frequently, also reported lower perceptions of harm.

This study corresponds with existing literature that with greater frequency of use, there is a decrease in harm perceptions [28]. Research has shown that decreased perceived risk is associated with increased engagement in the target health behavior [30] and that daily EC users find EC use less harmful than cigarettes [31]. Research has shown that compared to combustible cigarettes, ECs expose users to significantly lower levels of toxicants which may confer reduced long-term harm [32,33,34]. While previous studies have shown similar results [20,21,28,30,31], it is important to note that this study examines JUUL use among a large sample of adult users, at a time when ECs remain minimally regulated.

Interestingly, JUUL was rated as less harmful than almost all other products, much like other EC studies comparing harm perceptions of ECs and other tobacco products [9,25]. However, JUUL was not rated as less harmful than cigalike, tank, or modified ECs contrary to our hypotheses. In addition, regression analysis results corresponded with literature showing that lower risk perceptions of ECs were associated with use of ECs [35,36]. Past research supports this and suggests that reduced harm perceptions are often correlated with general use of those products [28,36,37] and can predict subsequent use [38,39]. Interestingly, our sample included not only users, but triers of the product as well. For public health practitioners, it would be useful to develop public health interventions that are strong enough to prevent tobacco naïve individuals from using the product, while at the same time accurately describing the actual harm of JUUL. It is important to note that the actual harm of JUUL, particularly in the long-term, is not fully known, however e-cigarette aerosols, similar to JUUL, have been shown to lead to inhalation of heavy metals, volatile organic compounds, and flavoring additives [40]. One other study has also shown that formaldehyde and total aldehyde yields were lower in JUUL compared to other studied ECs, but JUUL also had a very high nicotine content, which could lead to heavy exposure of the toxicants and addictive nicotine [17]. However, further research is warranted in this area.

Lastly, JUUL was rated as more harmful among women than men. This coincides with prior research suggesting that women are more risk averse than men [41]. Based on the results of this study, interventions and public health campaigns should focus on targeting specific groups of users possibly based on sex.

While the present study provides key insights into perceptions and associations of JUUL and JUUL use status, it is not without limitations. First, data were collected online using a convenience sample, and thus cannot be generalized to the population of EC users over 18 years old. MTurk samples can differ demographically, especially as it pertains to household income in addition to having a higher prevalence of EC use [42]. Second, data were collected prior to the FDA’s actions and JUUL self-regulation aimed at limiting youth (<21 years old) access to flavors other than menthol, mint, and tobacco at convenience marts and gas stations. Therefore, user perceptions and use of JUUL may have shifted following these regulatory actions. Future research incorporating how these new regulatory efforts may have adjusted perceptions of harm and use patterns are needed. Third, this study did not assess perceptions among those who have never tried JUUL or those who use another e-cigarette that is not JUUL. Fourth, this study did not assess comparative harm and only compared absolute harm among products. Future studies examining this association should ask participants about comparative harm and should consider more robust measures of harm and risk perception, including nuances, such as harm associated with frequency of use and harm to self versus others [43]. Lastly, it is important to acknowledge the correlational nature of the study. Longitudinal research is needed to show whether reduced harm perceptions lead to use of the product or if use of the product leads to reductions in harm perceptions.

## 5. Conclusions

Nevertheless, this study provides some of the first evidence of the associations between decreased perceptions of harm and more frequent JUUL use in a sample of adult ever-users. Given the new regulation restrictions on JUUL and the development of new pod-mod devices similar to JUUL, more research is needed to determine how perceptions are developed among adult users.

## Figures and Tables

**Table 1:** Frequency of JUUL use and other tobacco products.

Variables	Never User		Daily		Non-Daily		Trier	
	*N*	%	*N*	%	*N*	%	*N*	%
JUUL	0	0.0%	90	10.8%	246	29.4%	501	59.9%
Cigalike	165	20.0%	46	5.6%	149	18.1%	464	56.3%
Tank	201	24.7%	85	10.5%	134	16.5%	393	48.3%
Mod	276	34.2%	93	11.5%	101	12.5%	338	41.8%
Cigarette	144	17.4%	244	29.5%	112	13.5%	327	39.5%
Cigar	507	61.0%	7	0.8%	36	4.3%	281	33.8%
Cigarillos	556	67.3%	15	1.8%	38	4.6%	217	26.3%
Little Cigar	682	82.7%	11	1.3%	28	3.4%	104	12.6%
Pipe	711	85.6%	10	1.2%	12	1.4%	98	11.8%
Waterpipe	421	50.6%	7	0.8%	66	7.9%	338	40.6%
Smokeless Tobacco	580	70.1%	25	3.0%	31	3.7%	191	23.1%
Snus	685	83.2%	5	0.6%	30	3.6%	103	12.5%

Note. (1) never user, if the participant had never tried the product, (2) daily, if the participant used the product daily, (3) non-daily, if the participant used the product between once a week and once a month, and (4) trier, if the participant used the product less than monthly or not at all.

**Table 2:** Differences in absolute harm perceptions of JUUL vs. all other tobacco products.

Variables	N	Mean	Std. Deviation	Mean Difference from JUUL	*p*
JUUL	839	5.42	2.850		
Cigalike	839	5.63	2.793	−0.215	0.924
Tank	839	5.53	2.779	−0.108	1.000
Mod	839	5.44	2.781	−0.023	1.000
Cigarette	839	8.74	1.997	−3.324 *	<0.001
Cigar	839	7.81	2.473	−2.389 *	<0.001
Cigarillos	839	7.80	2.533	−2.379 *	<0.001
Little cigar	839	7.85	2.481	−2.433 *	<0.001
Pipe	839	7.85	2.457	−2.431 *	<0.001
Waterpipe	839	6.65	2.695	−1.234 *	<0.001
Smokeless tobacco	839	8.14	2.376	−2.721 *	<0.001
Snus	839	7.98	2.409	−2.567 *	<0.001
Dissolvable tobacco	839	6.64	2.785	−1.219 *	<0.001
NRT	839	4.72	3.079	0.692 *	<0.001

Note. One-way between subjects’ ANOVA with a Tukey post-hoc test adjustment for multiple comparisons. * Indicates significance. NRT is nicotine replacement therapy.

**Table 3:** Regression model of perceived harm of JUUL.

Variables	Unstandardized Coefficients		Standardized Coefficients	*t*	Sig.
	B	Std. Error	Beta		
(Constant)	3.96	0.48	-	10.94	<0.001
Female	0.42	0.20	0.07	2.14	0.03
Age	0.02	0.01	0.06	1.83	0.07
Race ^a^					
Black	0.21	0.32	0.02	0.67	0.51
Other/More than One	−0.29	0.29	−0.03	−1.01	0.32
Current use ≥1 product other than JUUL	−0.50	0.21	−0.08	−2.32	0.02
JUUL Use ^b^					
Non-Daily	0.75	0.35	0.12	2.15	0.03
Triers	1.24	0.32	0.21	3.83	<0.001

Note. ^a^. White = reference, ^b^. Daily use of JUUL = reference; Harm perceptions were assessed with the question, “On a scale from 0 to 10, how harmful are e-cigarettes?” where 0 was “not harmful at all” and 10 was “extremely harmful”.
